# *Saccharomyces cerevisiae*-Based Probiotics as Novel Antimicrobial Agents to Prevent and Treat Vaginal Infections

**DOI:** 10.3389/fmicb.2020.00718

**Published:** 2020-04-21

**Authors:** Roberta Gaziano, Samuele Sabbatini, Elena Roselletti, Stefano Perito, Claudia Monari

**Affiliations:** ^1^Department of Experimental Medicine, University of Rome Tor Vergata, Rome, Italy; ^2^Department of Medicine, Medical Microbiology Section, University of Perugia, Perugia, Italy

**Keywords:** probiotics, *Saccharomyces cerevisiae*, vulvovaginal candidiasis, bacterial vaginosis, vaginal microbiota

## Abstract

Vaginal infections affect 70% of women during their lifetimes and account for millions of annual doctors’ visits. These infections are predominantly represented by vulvovaginal candidiasis (VVC) and bacterial vaginosis (BV). Although standard antimicrobial agents remain the major strategy for the prevention and treatment of vaginal infections, both VVC and BV are difficult to treat due to high rates of resistance and recurrence, high probability of complications, and negative effects on the vaginal microbiota. This review focuses on a new approach of yeast-based probiotics for the prevention and/or treatment of these common vaginal infections.

## Introduction

It is estimated that vaginal infections account for more than 10 million doctors’ visits per year, and that 70% of episodes of vaginitis in premenopausal women are caused by bacterial vaginosis (BV) or vulvovaginal candidiasis (VVC; Anderson et al., 2004). Especially if untreated, these infections may cause serious complications of the upper genital tract (e.g., endometritis, salpingitis, and pelvic inflammatory disease) leading to tubal scarring, infertility, or ectopic pregnancies (Mulu et al., 2015; Kaambo and Africa, 2017). During pregnancy, they can increase the risk of preterm labor and low birth weight in the newborn (Guaschino et al., 2006). Further, some vaginal infections are associated with cellular abnormalities of the lower genital tract, which can lead to the development of cervical or vulvar dysplasia (Ravel et al., 2011). To date, although standard therapeutic strategies have been shown to be effective, they are not resolute. The increased drug resistance in bacteria and fungi, commonly involved in vaginal infections is frequently associated with high recurrence rates and chronic infections. Furthermore, the extant antimicrobial therapeutic strategies cannot spontaneously restore the normal vaginal flora, which is characterized by a high population of *Lactobacillus* spp. (Nyirjesy, 2014). Based on these observations, novel antimicrobial approaches are required. This review focuses attention on the potential benefits of yeast probiotic-based strategies as novel prophylactic or therapeutic options for vaginal infections.

## Vaginal Microbiota

The vaginal ecosystem contains many microorganisms, both anaerobic and aerobic, which are in a state of dynamic equilibrium (Gajer et al., 2012). It is influenced by various factors such as age, sexual activity, pregnancy, contraception, phases of the menstrual cycle, and possible pathologies or therapeutic treatments. Recent studies have demonstrated that at least five vaginal community state types (CSTs) exist in women of healthy reproductive age (Ravel et al., 2011; Gajer et al., 2012), of which four are dominated by lactobacilli. In detail, CST-I is dominated by *Lactobacillus crispatus;* CST-II, by *Lactobacillus iners*; CST-III, by *Lactobacillus gasseri*; and CST-V, by *Lactobacillus jensenii.* CST-IV is defined as a “non-*Lactobacillus*-dominated” community, and consists of strict and facultative anaerobes belonging to the genera *Gardnerella*, *Atopobium*, *Mobiluncus*, *Corynebacterium*, *Peptococcus*, *Peptostreptococcus*, *Clostridium*, *Bifidobacterium*, *Propionibacterium*, *Eubacterium*, *Bacteroides*, and *Prevotella*.

Despite a wide body of knowledge about the composition of vaginal microbiota exists, little is known about the composition of the vaginal mycobiome. Recent studies (Guo et al., 2012; Bradford and Ravel, 2017) have reported that in healthy women, Ascomycota is the predominant phylum, followed by Basidiomycota and Oomycota. *Candida, Saccharomyces, Aspergillus, Cladosporium*, and *Alternaria* represent the commonly genera of the vaginal mycobiome (Guo et al., 2012; Drell et al., 2013). *Candida* is the most abundant and is present in approximately 20–30% of healthy women (Bradford and Ravel, 2017; Cauchie et al., 2017). A balanced local microbial community is essential for vaginal health. Any perturbation of the composition of the vaginal microbiota, particularly a reduction in the population of *Lactobacillus* spp., which is considered the main component of a healthy vaginal ecosystem, may predispose women to genital tract infections.

## Vaginal Infections

The most common forms of vaginal infections are represented by VVC and BV. In particular, VVC affects women of reproductive age, and is caused by fungi belonging to the genus *Candida.* Among *Candida* species, *Candida albicans* (*C. albicans*) is responsible for almost 80–90% of all cases of VVC. A minority of cases (10–20%) is caused by other *Candida* species, including *Candida glabrata*, *Candida tropicalis*, and *Candida krusei* (Sobel, 2007). *C. albicans* is a dimorphic fungus that can exist as both yeast and mold. In the yeast form, *Candida* can asymptomatically colonize the vaginal mucosa. However, under some circumstances, the yeast cells switch from the yeast to hyphae, which can breach mucosal surfaces, causing acute vulvovaginal infection. Furthermore, *C. albicans* is the main etiological agent responsible for a severe chronic infection known as recurrent VVC. Although over 50% of women develop VVC, approximately 8–10% of these women experience recurrences (Nyirjesy, 2014). VVC development is, usually, attributed to alterations in the delicate balance between *Candida* commensalism and the host environment in the vagina, caused by physiological or non-physiological changes. Several host-related and behavioral risk factors can predispose to VVC, including pregnancy, hyperglycemia, immunosuppression, antibiotic or glucocorticoid therapies, oral contraceptive use, intrauterine devices, and genetic predispositions. However, despite the increasing list of well-known risk factors, the role of the host response to the microorganisms in causing *Candida* vaginitis and recurrent episodes remains to be fully clarified (Goncalves et al., 2016).

Bacterial vaginosis is considered the most common form of vaginitis affecting fertile, premenopausal, and pregnant women (Anderson et al., 2004). BV is often grouped together with trichomoniasis, a sexually transmitted infection caused by the parasite *Trichomonas vaginalis*, even though there are major differences in the etiology, pathophysiology, and transmission implications between the two. BV is not a true infection, but it is considered a complex imbalance in the physiological vaginal flora, where the normal population levels of *Lactobacillus* spp. are reduced and replaced by some of the less dominant and potentially pathogenic microorganisms, such as *Gardnerella vaginalis, Atopobium vaginae, Mobiluncus* spp., *Bacteroides* spp., *Mycoplasma hominis*, and *Prevotella* spp. (Srinivasan and Fredricks, 2008; Turovskiy et al., 2011). Microbiologically, BV is characterized by depletion of hydrogen peroxide (H_2_O_2_)-producing lactobacilli with an overgrowth of anaerobic bacteria. Although the exact mechanisms and sequence of events leading to the infective process have not yet been fully elucidated, *Gardnerella vaginalis* is thought to be a key player in the pathogenesis of BV, since it provides the niche suitable for colonization by anaerobic bacteria, which are primarily responsible for the clinical symptoms of BV (Turovskiy et al., 2011; Jung et al., 2017). There is general consensus that BV is characterized by the presence of anaerobic polymicrobial biofilm, consisting mainly of *Gardnerella vaginalis* (Swidsinski et al., 2008). Important features associated with the pathogenesis of this disease are: (i) the ability of *Gardnerella* to strongly adhere to vaginal epithelium forming a robust biofilm, (ii) the capacity to produce sialidase, an enzyme known to facilitate the destruction of the protective mucus on the vaginal epithelium, and (iii) the ability to trigger exfoliation of vaginal epithelial cells, which facilitates the spread of the pathogen to the underlying tissues.

Several studies have reported that multiple risks can predispose a woman to BV acquisition, including racial characteristics (black), smoking, low socioeconomic status, the presence of sexually transmitted diseases (STDs), and sexual behaviors (Marrazzo et al., 2010; Onderdonk et al., 2016). A growing body of evidence indicates that BV is consistently associated with an increased risk of STDs by parasites, like *Trichomonas vaginalis*, bacteria such as *Neisseria gonorrhoeae* and *Chlamydia trachomatis* (Allsworth and Peipert, 2011), and viruses including human immunodeficiency virus (HIV; Cu-Uvin et al., 2001; Cohn et al., 2005) and herpes simplex virus type 2 (HSV-2; Cherpes et al., 2008). The increased risk of acquiring STDs may further contribute to damage to the female reproductive organs.

## Conventional Antimicrobial Therapy

Several strategies have been employed in the clinical setting for the treatment of VVC. Standard therapy involves topical application of vaginal ovules, creams, lotions, or oral drugs. Fluconazole, amphotericin B, nystatin, and flucytosine are the most common antifungal agents currently in use to treat VVC. Among these, topical azole and oral fluconazole are equally efficacious and remain the first-line therapy for managing uncomplicated cases of VVC. In chronic or recurrent cases of vulvovaginitis, the first choice of therapy is oral fluconazole (Drago et al., 2017). Although the standard treatment for VVC leads to relief of symptoms and negative cultures in 80–90% of patients (Sherrard et al., 2011), the main limitation of the current antifungal treatments is their inability to offer a long-term defensive barrier (Sobel, 2016), facilitating relapses and a high recurrence rate. Possible mechanisms underlying recurrent VVC include genetic factors, reinfection from a sexual partner or from an endogenous source (the gut), persistence of fungi following treatment, probably due to emergence of resistant strains on the vaginal epithelium, and vaginal recolonization failure by *Lactobacillus* spp. (Nyirjesy, 2001; Liu et al., 2013; Sobel, 2016).

The therapeutic strategies recommended for BV treatment include (Paavonen and Brunham, 2018) oral or intravaginal metronidazole, or intravaginal 2% clindamycin cream (Centers for Disease Control and Prevention, 2015), and alternatively, oral administration of clindamycin and tinidazole (Livengood et al., 2007). In 2017, the FDA approved the use of secnidazole, a second-generation 5-nitroimidazole agent with a longer half-life than metronidazole and tinidazole for the treatment of BV (Sobel et al., 1993; Thulkar et al., 2012). Until now, although the current therapies initially succeed in reducing the symptoms of BV, extended follow-up studies have reported recurrence rates in excess of 50% within 6–12 month from the onset of therapeutic treatment (Bradshaw et al., 2006).

One possible reason for the failure of conventional treatments, and the high recurrence rates of BV, could be the strong polymicrobial biofilm that has been reported to be present in 90% of women with BV (Swidsinski et al., 2008) and the high levels of drug-resistance found in *Gardnerella vaginalis* clinical isolates (Gottschick et al., 2016). Alves et al. (2014) determined the *in vitro* susceptibility of 30 BV-associated biofilm-forming bacteria to metronidazole, tinidazole, and clindamycin and showed that all tested strains were resistant to metronidazole, and 67% of the tested strains were resistant to clindamycin. In addition, recent metagenome sequencing studies have identified at least four clades of *Gardenerella vaginalis* (Ahmed et al., 2012), of which two may be intrinsically resistant to metronidazole, providing another possible explanation for the BV persistence, even after appropriate therapies (Schuyler et al., 2016).

Therefore, it is evident that despite the use of standard antimicrobial agents, which remains the major strategy for treating vaginal infections, the high resistance and recurrence rates, the high probability of complications, and several adverse effects on beneficial vaginal microbiota underscore the need for novel antimicrobial therapeutic approaches.

## Yeast-Based Probiotics as a Novel Therapeutic Approach

In recent years, probiotic-based strategies have shown to be a promising and valid tool for both prophylaxis and treatment of vaginal infections both as alternative or adjunctive treatment. According to the definition of the Food and Agriculture Organization/World Health Organization, probiotics are defined as “Live microorganisms that, when administered in adequate amounts, confer a health benefit on the host” (Hill et al., 2014). Notably, a probiotic must to be safe for use in humans, and it must be effective, stable, and not be a carrier of acquired and/or transmissible antibiotic resistance. Therefore, a new assessment of safety, functionality, stability, and antibiotic resistance profile would be required before using any new microbial strain, even if it is one belonging to a species already in use. In addition, as the antimicrobial effect of a probiotic is generally strain-specific as well as disease-specific (Hasslof et al., 2010), a potential probiotic should be specifically chosen for a pathogen and disease and tailored to the individual patient. Probiotics can be used both orally or topically to prevent or treat vaginal infections, and currently neither route has a clear superior effect to the other. A pharmaceutical preparation of yeast-based probiotics for local treatment in different mucosal niches would be desirable.

Thus far, most microorganisms, used as probiotics, are cultivable components of the human microbiota and belong to the genera *Lactobacillus*, *Bifidobacterium*, and *Saccharomyces* (Amara and Shibl, 2015), which are included in the category of “generally regarded as safe” (GRAS; Kligler and Cohrssen, 2008; Snydman, 2008).

Over the last few years, interest around the use of yeast-based probiotics is increased, because not only they are naturally resistant to antibiotics, and, so, it’s not necessary to evaluate their antibiotic resistance profile, but, also, because they can be used in patients undergoing antibiotic therapy. Due to these characteristics they provide a considerable advantage over bacterial-origin probiotics. Although many yeast species have been shown to possess the characteristics of a probiotic, such as *Kluyveromyces lodderae, Kluyveromyces marxianus* (Kumura et al., 2004), *Kluyveromyces lactis*, *Yarrowia lipolytica* (Li-Shui et al., 2010), and *Issatchenkia occidentalis* (Kunyeit et al., 2019). *Saccharomyces boulardii* and *Saccharomyces cerevisiae* undoubtedly display the most probiotic properties. *S. boulardii* CNCM I-745 was the first strain that has been studied for use as probiotic in human medicine, and it is one of the recommended probiotics for the prevention and treatment of antibiotic-related diarrhea, including *Clostridium difficile-*associated diarrhea (Czerucka and Rampal, 2019). Additionally, preclinical studies have shown that this *Saccharomyces* strain presents a beneficial effect against many gastrointestinal pathogens such as *Salmonella typhimurium*, *Shigella flexneri*, *Escherichia coli* (enteropathogenic and enterohaemorrhagic strains), *Vibrio cholerae*, *Rotavirus*, and *C. albicans* (Czerucka and Rampal, 2019; Sen and Mansell, 2020).

Many studies have, also, highlighted the beneficial effects of several *S. cerevisiae* strains on entheropatogenic bacteria (Martins et al., 2005; Perez-Sotelo et al., 2005; Martins et al., 2007; Etienne-Mesmin et al., 2011; Tiago et al., 2012; Sivignon et al., 2015; Roussel et al., 2018), on inflammatory bowel diseases (Pineton de Chambrun et al., 2015; Tiago et al., 2015; Spiller et al., 2016; Cayzeele-Decherf et al., 2017b; Gayathri et al., 2020), and on pathogenic fungi (*C. albicans* and non-*albicans Candida* species, *Aspergillus flavus*; Premanathan et al., 2011; Abdel-Kareem et al., 2019; Kunyeit et al., 2019; Roselletti et al., 2019b). The potential mechanisms described include: inhibition of pathogen growth (Etienne-Mesmin et al., 2011; Roussel et al., 2018; Abdel-Kareem et al., 2019; Roselletti et al., 2019b), inhibition of pathogen adherence to epithelial cells (Perez-Sotelo et al., 2005; Tiago et al., 2012; Sivignon et al., 2015; Roussel et al., 2018; Kunyeit et al., 2019), immunomodulatory activity (Martins et al., 2005; Martins et al., 2007; Sivignon et al., 2015; Tiago et al., 2015; Roussel et al., 2018; Roselletti et al., 2019b), inhibition of filamentation and biofilm development (Kunyeit et al., 2019), and reduction of toxin production (Roussel et al., 2018; Abdel-Kareem et al., 2019).

Recently we demonstrated, for the first time, that a *S. cerevisiae*-based approach may be a very promising and valid tool for both prophylaxis and/or treatment of vaginal diseases such as VVC (Cayzeele-Decherf et al., 2017a; Pericolini et al., 2017; Gabrielli et al., 2018) and BV (Sabbatini et al., 2018).

These studies were performed with a well-characterized *S. cerevisiae* strain (European Committee for Standardization, 2009) owned by Lesaffre International, registered in the French National Collection of Cultures of Microorganisms (CNCM) under the number I-3856.

## *Saccharomyces cerevisiae*-Based Probiotic Effects on Vaginal Candidiasis and Bacterial Vaginosis

It is well known that the use of wide-spectrum antibiotics, including antifungal agents, leads to deep alterations in the vaginal microbiota, favoring the onset of vaginal infections. Using an *in vivo* imaging system, we demonstrated, in a mouse model of VVC, that the daily intravaginal administration of live *S. cerevisiae* (CNCM I-3856 strain) and, to a lesser degree, of inactivated *S. cerevisiae* (CNCM 1-3856 strain), elicited *C. albicans* clearance at levels similar to those obtained with fluconazole (Pericolini et al., 2017), the conventional drug used to treat *Candida* vaginitis (Workowski, 2015; Workowski et al., 2015). The beneficial effect of both live and inactivated *S. cerevisiae* was due to a co-aggregation of *Candida* and consequently to its inability to adhere to the mucosal surface, protecting the vaginal epithelium from the fungus induced damage (Pericolini et al., 2017). However, only the live and not the attenuated yeast strongly suppressed some of the crucial virulence factors of *C. albicans*, such as its capacity to switch from the yeast to the hyphal form and the ability to express aspartyl proteases (Pericolini et al., 2017; Gabrielli et al., 2018). These effects were related to the ability of the live yeast to significantly inhibit the expression of two important hyphal growth-associated genes, in particular the hyphal wall protein 1 (HWP1) and extent of cell elongation 1 (ECE1), as well as the expression of two secretory aspartyl proteinases (SAPs), SAP2, and SAP6, which play a key role in the immunopathogenesis of vaginal candidiasis (Naglik et al., 2003; Cassone and Cauda, 2012; Gabrielli et al., 2015; Pericolini et al., 2015; Roselletti et al., 2017). The suppression of *SAP2* and *SAP6* gene expression mirrored the reduction of the inflammatory process associated with the pathogenesis of *Candida* vaginitis (Roselletti et al., 2017). Indeed, SAPs, through a direct chemotactic activity (Gabrielli et al., 2016), promote the massive recruitment of neutrophils to the vaginal compartment, contributing to exacerbate the pathological inflammation associated to *Candida* infection (Peters et al., 2014; Vecchiarelli et al., 2015). Additionally, in our experimental models, *S. cerevisiae* has been shown to influence the host immune response by increasing the antimicrobial property of polymorphonuclear (PMN) cells, in terms of reactive oxygen species (ROS) hyperproduction and killing activity (Gabrielli et al., 2018), which generally appears to be reduced or absent in patients with vaginal candidiasis (Yano et al., 2017). Since ROS play an important role in triggering the extracellular trap (Kenno et al., 2016), which is one of the main mechanisms by which neutrophils can kill non-phagocytosed microorganisms, our results suggest that *Saccharomyces* may be able to stimulate this process *via* increasing the ROS production.

Moreover, the intravaginal administration of the live CNCM I-3856 strain downregulated the production of interleukin (IL)-8, a neutrophil chemotactic factor, which plays a role in the pathogenesis of human VVC (Roselletti et al., 2017). As we have recently found that in patients with VVC high IL-8 levels in vaginal fluid are positively associated with *Candida* infection (Roselletti et al., 2019a), IL-8 downregulation may represent an intriguing strategy for treatment of *Candida* vaginitis. These findings are supported by recent clinical results showing that daily oral administration of 500 mg (5 × 10^9^ CFU/mL) of live CNCM I-3856, for 56 days, in women receiving conventional antifungal drugs for VVC, was effective in controlling the vaginal *C. albicans* load and, also, in preventing VVC recurrence (Cayzeele-Decherf et al., 2017a). These data confirmed the therapeutic activity of *S. cerevisiae* CNCM I-3856 and further highlight the effectiveness of the oral administration of this strain in treating VVC. Of note, no serious side effects were observed in CNCM I-3856 yeast treated patients, confirming the safety and well-tolerability of this probiotic (Pineton de Chambrun et al., 2015; Spiller et al., 2016; Cayzeele-Decherf et al., 2017a, b). However, future studies should be carried out for a better understanding of dynamics within *Saccharomyces* given orally can colonize the vaginal microenvironment.

Despite accumulation of data regarding the protective effect against mucosal candidiasis, no studies have investigated the impact of yeast-based probiotics in BV. Recently, our research group demonstrated, for the first time, that vaginal administration of *Saccharomyces* CNCM I-3856 strain, also, had beneficial effects in a mouse model of *G. vaginalis* infection (Sabbatini et al., 2018). Indeed, this strain significantly reduced the vaginal bacterial load and removed up to 90% of *Gardnerella* bacteria infecting uterine horns, suggesting that the CNCM I-3856 strain presents a potential therapeutic efficacy in treating not only vaginal candidiasis but, also, bacterial uterine infections. This is a noteworthy effect because several studies have identified *G. vaginalis* as an etiological agent in puerperal sepsis and in septic and endometritis abortion (Adeniyi-Jones et al., 1980; Reimer and Reller, 1984; Johnson and Boustouller, 1987). The efficacy of this strain was associated with a marked reduction of *Gardnerella* virulence factors, including sialidase activity and inhibition of vaginal epithelial cells exfoliation. Additionally, mechanistic effects include the direct interference of CNCM I-3856 yeast with *Gardnerella* adherence to vaginal tissues, and its ability to exert a displacement of adhering bacteria to vaginal or cervical epithelial cells. Table 1 summarizes the beneficial effects of *S. cerevisiae* CNCM I-3856 in the prevention and treatment of vaginal infections.

**TABLE 1:** Effect of *S. cerevisiae* CNCM I-3856 on vaginal infections.

*S. cerevisiae* CNCM I-3856 effects on VVC	References
Reduction of *C. albicans* vaginal load	Cayzeele-Decherf et al., 2017a; Pericolini et al., 2017
Inhibition of *C. albicans* adhesion on vaginal epithelial cells	Pericolini et al., 2017
Induction of *C. albicans* co-aggregation	Pericolini et al., 2017
Suppression of *C. albicans* ability to switch from yeast to hyphal form	Pericolini et al., 2017
Suppression of *C. albicans SAP2* and *SAP6* expression	Pericolini et al., 2017; Gabrielli et al., 2018
Reduction of vaginal epithelial cell damage	Pericolini et al., 2017
Reduction of IL-8 production. Reduction of PMNs vaginal recruitment	Gabrielli et al., 2018 Gabrielli et al., 2018
Enhancement of PMNs antimicrobial activity	Gabrielli et al., 2018

***S. cerevisiae* CNCM I-3856 effects on BV**	**References**

Reduction of *G. vaginalis* vaginal and uterine horns load	Sabbatini et al., 2018
Inhibition of *G. vaginalis* adhesion on vaginal epithelial cells	Sabbatini et al., 2018
Displacement of *G. vaginalis* adhered to vaginal epithelial cells	Sabbatini et al., 2018
Reduction of *G. vaginalis* sialidase activity	Sabbatini et al., 2018
Reduction of vaginal epithelial cells exfoliation	Sabbatini et al., 2018

## Conclusion and Future Perspectives

Vulvovaginal candidiasis and BV are the most prevalent vaginal infections in women, and both are characterized by an extremely high recurrence rate mostly due to the emergence of resistant strains against the commonly used antimicrobial drugs. Probiotic administration could represent an alternative or adjuvant therapeutic approach for preventing and/or treating VVC and BV. Despite several preclinical researches and a multitude of clinical trials, to date, few studies have analyzed the effects of yeast-based probiotics on vaginal infections. This is the first overview of the beneficial effects of a probiotic yeast in preventing and/or treating some vaginal mucosal infections. Local and/or oral treatment with *S. cerevisiae* attenuated the course of VVC, as well as, BV in a mouse experimental system. The positive effect of this treatment was confirmed with a controlled clinical trial in women with vaginal candidiasis. The mechanism appears to be associated with a direct effect of *S. cerevisiae* on pathogens, as well as, in the case of VVC, its immunobiotic properties. These features open the door to future clinical studies to determine if *S. cerevisiae* CNCM I-3856 can reduce the colonization of *C. albicans* and/or *G. vaginalis* on human mucosal surfaces, attenuate VVC and/or BV symptoms, and enhance the antimicrobial effect of standard therapeutic approaches.

## Author Contributions

CM conceived, planned, wrote, and revised the manuscript. RG planned, wrote, and revised the manuscript. SS, ER, and SP wrote and revised the manuscript. All authors have read and approved the final manuscript.

## Conflict of Interest

The authors declare that the research was conducted in the absence of any commercial or financial relationships that could be construed as a potential conflict of interest.
